# How to Improve the Well-Being of Youths: An Exploratory Study of the Relationships Among Coping Style, Emotion Regulation, and Subjective Well-Being Using the Random Forest Classification and Structural Equation Modeling

**DOI:** 10.3389/fpsyg.2021.637712

**Published:** 2021-04-26

**Authors:** Xiaowei Jiang, Lili Ji, Yanan Chen, Chenghao Zhou, Chunlei Ge, Xiaolin Zhang

**Affiliations:** ^1^Institute of Psychology and Behavior, Henan University, Kaifeng, China; ^2^Institute of Cognition, Brain and Health, Henan University, Kaifeng, China

**Keywords:** subjective well-being, emotion regulation, coping style, random forest (bagging) and machine learning, structural equation model

## Abstract

The relationship between coping styles and subjective well-being (SWB) has recently received considerable empirical and theoretical attention in the scientific literature. However, the mechanisms underlying this relationship have primarily remained unclear. The present research aimed to determine whether emotion regulation mediated the relationship between coping styles and subjective well-being (SWB). Our hypothesis is based on the integration of theoretical models among 1,247 Chinese college students. The SWB questionnaire, Ways of Coping Questionnaire, and Emotion Regulation Questionnaire were used to correlate SWB, emotion regulation strategies, and coping styles, respectively. The random forest method was applied to predict life satisfaction and estimate the average variable importance to life satisfaction. The results indicated that positive coping can indirectly influence life satisfaction via cognitive reappraisal and indirectly influence expression suppression via positive affect. Negative coping can indirectly influence negative affect via expression suppression. Besides, negative coping was positively associated with both expression suppression and negative affect. Cognitive reappraisal was found to be positively associated with positive affect. The findings indicated that coping style is essential for the SWB of college students. These findings provide insight into how coping styles impact SWB and have implications for developing and assessing emotion regulation-based interventions.

## Introduction

Subjective well-being (SWB) is an overall emotional and cognitive evaluation that individuals make about the quality of life, which consists of life satisfaction, positive affect, and negative affect (Diener and Emmons, 1984; Diener et al., 1999; Keyes et al., 2002). Research indicates that SWB has merits for mental health and longevity, supportive social relationships, citizenship, work performance, and resilience (De Neve et al., 2013).

Under globalization, the social development process is speeding up, thus increasing pressure and competition of individuals, as well as college students. With the increasing pressure coming from the study, employment, competition, and interpersonal relationship problems, college students' mental health will face a lot of new situations and new problems. And with the increase of all kinds of psychological pressure, the psychological problem appearing presents the trend of accelerating growth. Research showed that approximately half of mental health problems begin in midadolescence, whereas 75% of adult mental health problems start before the age of 24 years (Kessler et al., 2007; Jones, 2013). It has been reported that more than 20% of youth often suffer from mental health problems, such as anxiety, insomnia, and depression (Costello et al., 2003; Merikangas et al., 2010; Jones, 2013; Dooley et al., 2015; Achenbach et al., 2016). Consequently, it is a considerably overarching goal of education to explore how we can protect university students' mental health.

Lots of studies proved that SWB could be remarkably affected by objective factors, such as economic income, daily experience of life, cultural atmosphere, and internal factors, such as self-efficacy, coping style, and attributional style (Zhang et al., 2014). Although highly economically developed Western nations have presented many related demonstrations as SWB is highly valued in these prosperous nations, hardly do we gather the same knowledge of those applications in Asia, especially in China. In a very small number of studies, researchers have investigated the relationship between Chinese youths' friendship quality (Niu et al., 2021), mobile social networks (Hu and Guo, 2021), and SWB. The study of Hu and Guo (2021) aimed to inspect the relationship between interpersonal communication on mobile social networks and SWB. They found that interpersonal connection in the mobile social network has a significant predictive effect on SWB. Niu et al. (2021) explored the relationship between adolescents' friendship quality and SWB with the meta-analysis method. Results showed that adolescents' friendship quality had a positive correlation with SWB.

The occupation of SWB as a factor to influence mental and body health among Chinese University students is still unclearly understood in recent studies. And it provides a beneficial solution that improves the quality of the appropriate intervention of dealing with psychosocial issues of university students. Given the limitations of the empirical studies in psychological counseling, this investigation examined variables related to SWB in college students. It is specific that the relationships among SWB, emotional regulation strategies, and coping strategies are examined herein.

### SWB

SWB has been widely associated with happiness and hedonia. It includes reflective cognitive judgments, life satisfaction, and current emotional responses concerning positive and pleasant emotions versus unpleasant and negative emotions and extensively adopted the definition provided by Diener that by evaluating the whole life among various domains, e.g., health, work, family, income, or people's actual feelings, the feelings of individuals were split into positive feelings (e.g., delighted, blissful) and negative ones (e.g., disgruntled, pain) (Diener and Emmons, 1984; Diener et al., 1999). It has been considered that SWB consists of emotional states, global ratings of life satisfaction, and satisfaction with certain life domains (Eid and Larsen, 2008). These conceptualizations imply on the whole that SWB has both cognitive and emotional aspects. It is assumed that relatively stable and global evaluations of life circumstances can be reflected in cognition aspects. On the other hand, emotional well-being is assumed to be more sharply influenced by daily uplifts and hassles (Larsen and Prizmic, 2008). It is usually presented by the frequency and intensity of positive affect and negative affect. Affective and cognitive aspects in life are associated with a significant correlation between life satisfaction and positive affect instead of life satisfaction and negative affect (Lucas et al., 1996).

Nevertheless, research suggests that constructs among life satisfaction, positive affect, and negative affect are distinct. It has been shown that positive affect and negative affect are independent dimensions of emotion (e.g., Larsen and Prizmic, 2008). These various facets of SWB might cause a differential influence on health and longevity (Diener et al., 2017).

### Coping Styles

The coping strategy was defined by Lazarus and Folkman (1986), who identified it as an adaptive response to a specific stressor. Coping strategy is the process of dealing with stress among individuals, utilizing various psychological strategies and mechanisms (both cognitive and behavioral) to reduce, control, or tolerate it (Parker and Endler, 1992). Cognitive stress theory (Lazarus and Folkman, 1984) posits that appraisal and coping variables mediate individual differences in outcomes, such as valuations of quality of life. When the demands far overwhelm the available sources for individuals, they tend to merge available resources with suitable strategies to reduce stress and improve the overall quality of life. In this study, the interest is in determining which coping style can improve life quality.

A person's use of specific and planned techniques expressed in either cognitive or behavioral form can be reflected effectively by coping styles (Park and Iacocca, 2014). Specific coping strategies might adjust to life stress. Therefore, investigating specific coping styles (i.e., positive or negative) is critical to identify their relationship with the SWB.

Research shows that people with positive coping styles tend to use planning, seeking social support, positive restructuring, or problem solving (Doron et al., 2013; Kotze et al., 2013) to solve a stressful situation, thus improving SWB (Gustems-Carnicer and Calderon, 2013). Also, they have less likelihood of psychosocial disorders such as depression (Elliott et al., 1991), mental health symptoms, better well-being (Irving et al., 2004), and more positive consideration (Snyder et al., 1996). People with negative coping styles might avoid thinking about or suffering from the situation under pressure through rumination, self-blame, disengagement, or social isolation (Ryan and Deci, 2000; Tora et al., 2011; Kapetanovic et al., 2014). The negative coping style is useless in alleviating stress and prone to result in poor SWB (Anshel and Brinthaupt, 2014). Negative coping strategies are associated with depression, lower health, and SWB (Turner et al., 2000; Wollaars et al., 2007).

### Emotion Regulation

Emotion regulation has been defined as including all the conscious and unconscious strategies individuals use to reduce, maintain, or increase positive or negative emotions (Gross, 2001). Research has shown that various habitual uses of emotion regulation strategies cause multiple domains of psychological adjustments, such as affective experience and cognitive and social functioning (e.g., Gross and John, 2003; John and Gross, 2004). Similarly, emotion regulation problems play a prominent role in developing and maintaining psychopathological symptoms and emotional disorders (Gross and Munoz, 1995; Garnefski et al., 2002). Emotion regulation is embodied in an individual's capacity to regulate the potency, duration, and intensity of emotional experience (Gross, 1998; Gratz and Roemer, 2008). Among them, cognitive reappraisal and expression suppression are most valued by researchers. Cognitive reassessment is an antecedent-focused strategy to induce emotional regulation through subjective efforts to reinterpret the plot, which occurs at the preceding stage of emotion generation (Gross, 1998). Expression suppression is mainly reflected in the individual's behavior of suppressing the expression of emotion to regulate emotion consciously, which is a response-focused strategy and occurs at the late stage of emotion generation. Studies have found that cognitive reappraisal may help to maintain mental health (Gross and John, 2003; Hughes et al., 2011).

Several studies showed the relationship between habitual use of emotion regulation strategies and psychopathological symptoms, while far less attention has been paid to the impact of emotion regulation on well-being. For instance, Shiota (2006) found that positive reappraisal and creating positive sensory events have a more strongly correlation with SWB than seeking social support and problem-focused coping, while preceded by an adverse event, the habitual use of distraction was associated with the report of lower levels of well-being. Karademas (2007) found that higher well-being levels can be predicted by positive reappraisal and problem-focused positive coping predicted. On the contrary, negative coping did decrease well-being levels. Eventually, it bridges the gap among the habitual use of cognitive reappraisal, the experience of less negative affect, more positive affect, greater life satisfaction, and higher levels of psychological well-being (PWB) (Gross and John, 2003; Haga et al., 2009; McRae et al., 2012).

To date, most research has proven the possible relationship between emotion regulation and distress symptoms, thus identifying which strategies are disadvantageous (or protective) for emotional problems and can be essential targets of psychotherapy interventions (Garnefski and Kraaij, 2007). On the other hand, it is surprising to note that less research has examined the relationship between positive aspects of the individual's well-being with the individual's optimal psychological functioning and experience and the dispositional use of emotion regulation strategies in response to adversity (Folkman and Moskowitz, 2000; Ryan and Deci, 2001; Gross and John, 2003; Shiota, 2006; Karademas, 2007). It is a significant omission because ill-being and well-being can be deliberated as mostly independent, distinct domains of mental functioning, such that knowledge about the correlations of one does not necessarily extend to the other (Ryan and Deci, 2001). In other words, not only should well-being be conceived as the absence of psychopathology but also as for human strengths and potentials (Seligman and Csikszentmihalyi, 2000).

### The Present Study

The study aims to examine diverse coping styles, emotion regulation strategies, and SWB to determine the relationship among these variables. Measures of positive coping and negative coping determined evaluations of coping style. The habitual use of suppression and reappraisal were measured with the Emotion Regulation Questionnaire (ERQ). Positive affect, negative affect, and life satisfaction were indicators of SWB. All constructs were manipulated into scores for instruments with surpassing psychometric properties selected for their ability to measure a particular construct. Based on previous literature, it was hypothesized that (1) positive coping would be associated with cognitive reappraisal, positive affect, and SWB; (2) negative coping would be associated with expression suppression, negative affect, and SWB; and (3) emotion regulation would mediate the relationships between coping styles and SWB, life satisfaction, positive affect, and negative affect.

**Figure 1:**
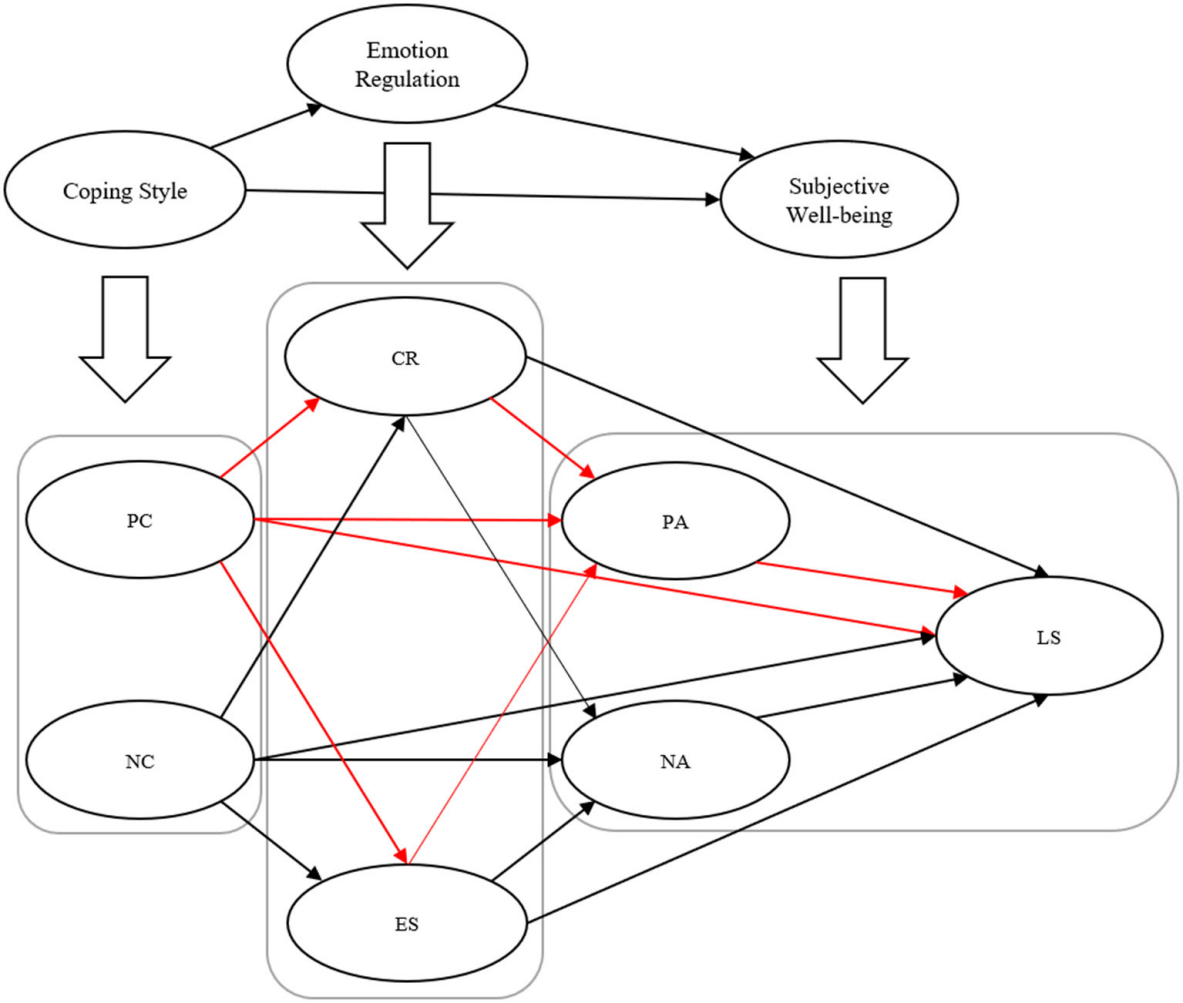
The proposed model in our study. Positive paths are marked in red. PC, positive coping; NC, negative coping; CR, cognitive reappraisal; ES, expression suppression; PA, positive affect; NA, negative affect; LS, life satisfaction.

This study is expected to provide a theoretical supplement to the research in related fields of positive psychology. Simultaneously, it can effectively guide and improve the SWB of college students and has particular practical guiding significance for promoting college students' mental health. The structure of the proposed model is shown in Figure 1.

## Materials and Methods

### Participants

The present study collected 1,247 college students in Henan University as a sample. One hundred nineteen participants with mistake values on more than four of ten lie detection items were excluded. Eventually, it left a final sample of 1,127 participants, and the average age was 20 years (SD = 1.199, range = 17–25 years). No participant reported any present or prior history of medical or psychiatric disorder.

### Measurement

Our study used a demographic questionnaire and two SWB measures [Satisfaction With Life Scale (SWLS; Xiong and Xu, 2009); Positive Negative Affect Schedule (PANAS; Huang et al., 2003)], a Ways of Coping measures [Ways of Coping Questionnaire (WCQ; Jie, 1998)], and an emotion regulation questionnaire [ERQ (Wang et al., 2007)].

#### SWB

The SWB consists of two subscales assessing Positive Affect and Negative Affect (Watson et al., 1988) and life satisfaction (Larsen et al., 1985). And the scale has good test–retest reliability and validity among Chinese college students.

The Chinese version of PANAS assesses positive and negative affect developed by Huang et al. (2003). It is a 7-point Likert-type measurement within 20 items, including two evaluation dimensions: positive affect (10 items) and negative affect (10 items), about how the subjects usually felt. The average scores of each dimension were computed, respectively. Positive and negative affect scales reached α coefficients of 0.902 and 0.890 each in the current study.

We used the SWLS (Chinese adaptation by Xiong and Xu, 2009) to evaluate life satisfaction. Five items measure the global life satisfaction. Respondents value their agreement using 7-point Likert-type scale items ranging from 0 (strongly disagree) to 6 (strongly agree). The score is computed by averaging the five items of the scale. High scores on the SWLS mean large life satisfaction. The α coefficient in the current sample was 0.836.

According to the confirmatory factor analysis (CFA), χ^2^ = 2219.259, degrees of freedom (*df*) = 272, χ^2^/*df* > 3, comparative fit index (CFI) = 0.869, root mean square error of approximation (RMSEA) = 0.080, in line with the above fitting indexes, the fitting indexes were good.

#### WCQ

WCQ is based on the original scale and combined with Chinese national conditions and actually needed to be revised (Jie, 1998). The scale consists of two dimensions: positive coping and negative coping. There are 20 questions in the whole scale, of which the positive coping dimension is composed of 1–12, and the negative coping dimension is composed of 13–20 (i.e., 0 = “do not use,” 1 = “occasionally use,” 2 = “sometimes use,” and 3 = “often use”). The retest correlation coefficient of the scale was 0.89, and Cronbach probability coefficient was 0.90. The Cronbach probability coefficient of actively responding to subscales was 0.80. That of the negative response subscale was 0.711, and that of the negative response subscale was 0.848 in this study.

#### ERQ

The Chinese version of the ERQ developed by Wang Li (Wang et al., 2007) was utilized to assess the emotion regulation strategies, including cognitive reappraisal and expression suppression (Gross and John, 2003).

The dimensions of cognitive reassessment included six items, and the dimensions of expression suppression included four items, among which the Cronbach probability coefficients were 0.834 and 0.731, respectively, and that in this study was 0.710.

### Data Analysis

First, we calculate descriptive statistics (mean and SD) of all four latent constructs' variables followed by Pearson correlation analysis between all indicator variables. All the variables in the measurement model were normally distributed.

Machine learning algorithms were then trained to fit this classification based on the predictive variables autonomously. And the random forest regression and classification model, one of the most popular bagging ensemble machine learning algorithms (Pedregosa et al., 2011), were trained to be compared to the performance. The random forest regression score was MAE (mean absolute error), which was used to describe the error between the predicted value and the real value.

$$\text{MAE}\left(\text{X},\text{h}\right)=\frac{\text{1}}{\text{m}}\sum_{\text{i=1}}^{\text{m}}\left|\text{h}\left({\text{x}}^{\left(\text{i}\right)}\right)-\left({\text{y}}^{\left(\text{i}\right)}\right)\right|$$

In classification model training, the life satisfaction was split into a new binary variable as two categories with values “low life satisfaction” (or “0”) if the life satisfaction score was <17 (620 samples), and “high life satisfaction” (or “1”) otherwise (507 samples). The prediction error is selected to be at the top of the tree, and it is split into two parts (for a continuous variable, a cutoff point is created using minimizing prediction error). This partitioning continues recursively to generate trees. Prediction is then made using averaging the response variable in each leaf of the final tree (in regression setting). The random forest technique applies random selection in two ways to improve the prediction performance of the tree regression methodology. In this regard, trees are formed by using random samples selected from all original observations (bootstrapped sampling) and a stochastic sample of candidate variables (inputs) for splitting (Breiman, 2001; Grömping, 2009). This randomness handles these trees' instability because it introduces differences in individual predictions obtained from each tree (Barnett et al., 1989). The averaging rule is used for all individual trees' results to obtain predictions for the final forest (Barnett et al., 1989; Breiman, 2001). And we also use tree-based boost classification models, such as Adaboost (Yoav and Robert, 1997), XGBoost (Tianqi and Carlos, 2016), LightGBM (Guolin et al., 2017), and Catboost (Anna et al., 2017; Liudmila et al., 2019), to compare with the random forest classification (RFC) model.

**Figure 2:**
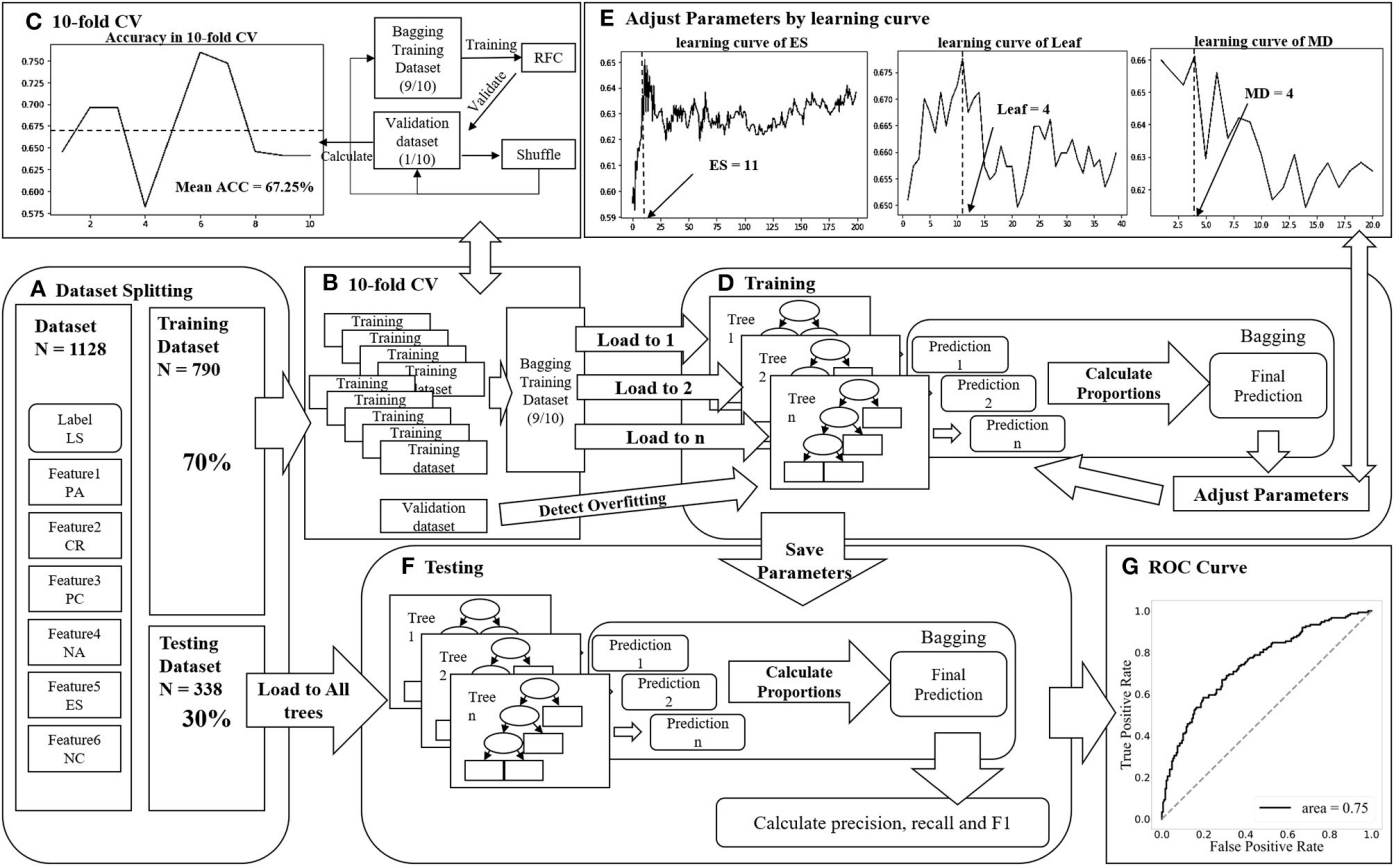
**(A)** Dataset was divided into a training dataset (70%) and a testing dataset (30%). PC, positive coping; NC, negative coping; CR, cognitive reappraisal; ES, expression suppression; PA, positive affect; NA, negative affect; LS, life satisfaction; RFC, random forest classification; ROC, receiver operating characteristic. **(B)** Training datasets were divided into 10 folds, one used to validate the model and the rest bagging to train a RFC model. **(C)** Tenfold cross-validation was reused 10 times to calculate the mean accuracy, and each time we randomly reshuffled our training folds and validation fold. **(D)** Many different decision trees were trained in RFC model by adjusting parameters to find the optimal fitting by training dataset and detect whether there was overfitting by validation dataset. **(E)** Learning curves were used to detect the optimal pairs of parameters. The accuracy of the model was calculated by the remaining other parameters and changing one parameter. After calculating all accuracies, we choose the value that presents the enormous accuracy. **(F)** After training, we use the testing dataset to calculate the trained RFC model's final index. **(G)** The ROC curve of RFC model shows the performance of this model.

After preprocessing, the current dataset was split stochastically into two parts, a training set to train the machine learning algorithms (70%) and testing (30%) as an independent test set to verify the final model. Subsequently, for assessing the trained machine learning algorithm based on each dataset, selecting one-tenth of the training set was randomly reserved as a testing set while remaining used for training repeated 10 times, depending on 10-fold cross-validation, thus calculating the overall average scales of this testing set as the original training scales. The independent test set verified its ensembling and obtained the independent test set results among all 10 models. A schematic diagram of RFC is presented in Figure 2.

We measured the feature importance according to the mean decrease of the Gini Index, which indicates the purity of a dataset's partition. The trees from this random forest have a specific variable of splitting a node before averaging the Gini Index's decrease. The higher the variable had, the more important it was considered. Variable importance was calculated based on the training set. After that, we used the SHapley Additive exPlanation (SHAP) model (Lundberg and Lee, 2017) to interpret the model and justify the contribution of each feature.

Finally, we test the hypothesized mediation model that specifies the relationship among SWB, coping styles, and emotion regulation by structural equation modeling, which involved a two-step process. The first phase was a measurement model named CFA to verify the latent constructs and their indicator variables' hypothesized relationships using standardized factor loadings. Next is a structural model, which was constructed to test the hypothesized relationships between all latent constructs. We estimated the standardized regression coefficients for all paths as we hypothesized by the maximum likelihood method of parameter estimation and covariance matrix in the R's lavaan package. The goodness-of-fit index (GFI), χ^2^ to *df* ratio, RMSEA, non-normed fit index (NNFI), and Bentler's CFI are useful indexes to fit and evaluate (Hatcher, 1994). Good model fit is determined by an RMSEA of <0.08 and values of GFI, CFI, and NNFI >0.90 (Hatcher, 1994; Hu and Bentler, 1999). To explore whether the effect of coping styles on SWB was mediated by emotion regulation, we tested the mediation effect using the regression analysis. There were no more than 2% of any variables as missing data, which were imputed using the mean replacement method in this study. All the data management and statistical analyses were performed using SPSS 25 and R3.6.1. The SEM was established in the lavaan package of R.

## Results

### Preliminary Analyses

Table 1 shows the descriptive statistics and correlations and indicates that the SWB was significantly correlated with both ways of coping and emotional regulation in all individuals. Some correlation coefficients were not significant. However, considering Simpson's paradox, there might be inhibitory variables. The relationship between variables could not be confirmed by correlation coefficients alone. Hence, our study used more complex measurements to fit our dataset, thus detecting the possible results.

**Table 1:** Means, standard deviations, and correlation for all variables (*N* = 1,128).

	**Mean (SD)**	**1**	**2**	**3**	**4**	**5**	**6**	**7**	**8**	**9**
**WC**										
1. PC	2.94 (0.5)	—								
2. NC	2.27 (0.6)	0.142^**^	—							
3. SWB	23.35 (13.4)	0.499^**^	−0.064^*^	—						
4. PA	29.02 (7.6)	0.439^**^	0.115^**^	0.605^**^	—					
5. NA	22.88 (7.8)	−0.172^**^	0.247^**^	−0.545^**^	0.234^**^	—				
6. LS	17.21 (5.8)	0.346^**^	0.030	0.790^**^	0.397^**^	−0.231^**^	—			
7. ER	33.51 (4.9)	0.253^**^	0.067^*^	0.178^**^	0.127^**^	−0.064^*^	0.160^**^	—		
8. CR	22.19 (3.8)	0.477^**^	0.016	0.377^**^	0.269^**^	−0.175^**^	0.286^**^	0.775^**^	—	
9. ES	11.33 (3.1)	−0.181^**^	0.087^**^	−0.177^**^	−0.126^**^	0.111^**^	−0.095^**^	0.640^**^	0.01	—

*WC, way of coping; PC, positive coping; NC, negative coping; SWB, subjective well-being; PA, positive affect; NA, negative affect; LS, life satisfaction; ER, emotion regulation; CR, cognitive reappraisal; ES, expression suppression*.

* *p < 0.05*,

** *p < 0.01*.

### Random Forest

First, we conducted exploratory data analysis to describe the distribution of each variable in the training and testing dataset, illustrated in Figure 4A. Two-way repeated-measures analysis of variance was adopted to evaluate the difference of datasets (train and test dataset) and samples (low-LS and high-LS samples). As shown in Figure 4B, there was no significant difference between train and test dataset in all negative affect, cognitive reappraisal, expression suppression, positive coping, and negative coping, whereas there were striking differences in negative affect, cognitive reappraisal, expression suppression, and negative coping between low-LS and high-LS samples. The MAE of RF regression was 0.213 and 0.177 on the training dataset and test dataset, respectively. The poor performance after training was due to the noise of the questionnaire dataset. Then, the life satisfaction was converted from continuous data into high life satisfaction and low life satisfaction labels according to the median. The random forest model provided by the scikit-learning package based on Python3.8 is used for training (Pedregosa et al., 2011). And the receiver operating characteristic curve and area under the curve of each model are shown in Figure 4C.

**Figure 3:**
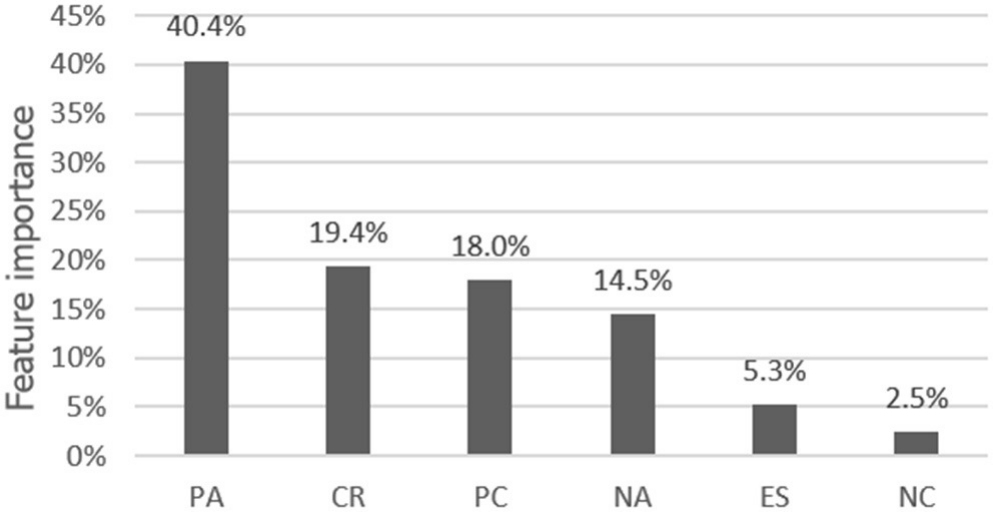
The average feature variable importance to life satisfaction: according to the mean decrease of the Gini Index. PC, positive coping; NC, negative coping; CR, cognitive reappraisal; ES, expression suppression; PA, positive affect; NA, negative affect.

Table 2 shows the results from the classification model of these two datasets in a different class. The performance of RFC was better than others. According to Gini Index's mean decrease, the random forest's variable importance is presented in Figure 3.

**Table 2:** Precision, recall, and F1 in testing procession in tree-based model.

**Test precession**	**Class**	**Precision**	**Recall**	**F1**	**Support**
RFC testing	Low LS	0.73	0.71	0.72	188
Accuracy = 0.69	High LS	0.65	0.67	0.66	151
	Macro	0.69	0.69	0.69	
	Weighted	0.69	0.69	0.69	
CatBoost testing	Low LS	0.69	0.8	0.74	188
Accuracy = 0.69	High LS	0.69	0.56	0.62	151
	Macro	0.69	0.68	0.68	
	Weighted	0.69	0.69	0.69	
AdaBoost testing	Low LS	0.72	0.72	0.72	188
Accuracy = 0.69	High LS	0.65	0.66	0.65	151
	Macro	0.69	0.69	0.69	
	Weighted	0.69	0.69	0.69	
XGBoost testing	Low LS	0.72	0.71	0.71	188
Accuracy = 0.68	High LS	0.64	0.65	0.65	151
	Macro	0.68	0.68	0.68	
	Weighted	0.68	0.68	0.68	
LightBoost testing	Low LS	0.73	0.71	0.72	188
Accuracy = 0.70	High LS	0.65	0.67	0.66	151
	Macro	0.69	0.69	0.69	
	Weighted	0.69	0.69	0.69	

*LS, life satisfaction*.

Accordingly, all the classification's performance is similar in this dataset. And the random forest model gets the highest recall in the high-LS class. Macro refers to the result of the arithmetic average of different indicators, and weighted refers to the weighted average of different indicators according to the sample size. There is not a dramatic difference between them. Consequently, it is basically not affected by the sample size. Using this random forest model to predict mathematical results can achieve a precision rate of ~68%, whereas the average F1, recall rate, and accuracy rate are all >60%, and the recall rate of only high life satisfaction is <60%. The prediction result of the high life satisfaction is slightly weaker than that of the low life satisfaction. And it can reach an average F1 of ~68%, a precision rate of ~68%, and a recall rate of ~68%. This result indicates that the discriminant model established by machine learning is acceptable.

**Figure 4:**
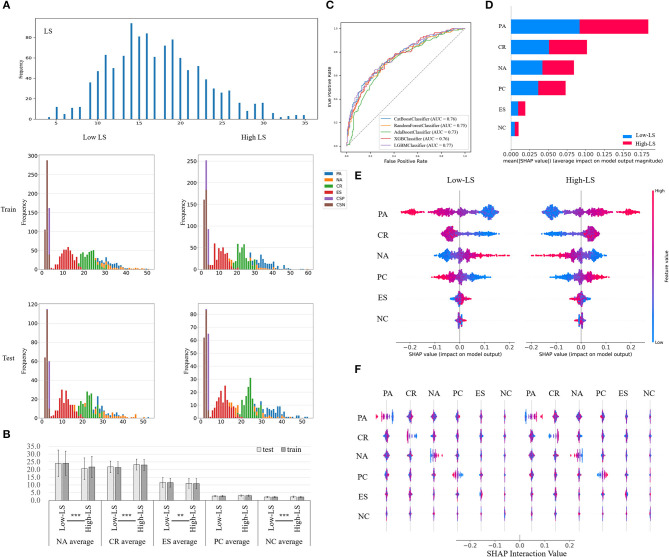
**(A)** This figure shows the result of the distribution of each feature selected. **(B)** It shows the average value in each group of the datasets. ^**^p < 0.01, ^***^p < 0.001. **(C)** It shows the ROC curve and AUC of each model. **(D)** It shows the average impact of each feature within predicting two classes of LS. **(E)** The color and *x*-axis index the feature value and SHAP value, respectively, among two classes (low LS and high LS). **(F)** It shows the interaction of each variable among two classes (low LS and high LS). AUC, area under the curve; PC, positive coping; NC, negative coping; CR, cognitive reappraisal; ES, expression suppression; PA, positive affect; NA, negative affect; LS, life satisfaction; ROC, receiver operating characteristic.

Using SHAP (based on the package named shap in Python) to explain the classification model, the results showed that the importance of each feature calculated by SHAP is similar to the ones by the Gini Index in Figures 3, 4D,E, and the most important feature is still positive affect. After that, we also presented the interaction relationship of each feature in Figure 4F.

### Regression Analysis

According to the hypothesis, the mediation effect of emotion regulation is verified through the regression analysis. The results are shown in Table 3; all coefficients are significant in the table, and the two coping styles among all individuals to two types of emotion regulation and SWB of the four paths partial mediation effect are established. However, it is unable to determine the interaction relationship between the paths and requires further analysis.

**Table 3:** Parameter description of variables in regression analysis.

**Dependent variable**	**Independent variable**	**Standardized β**	***p***
SWB (*R*^2^ = 0.298)	PC	0.41	0.000^***^
	NC	−0.12	0.000^***^
	CR	0.19	0.000^***^
CR (*R*^2^ = 0.230)	ES	−0.09	0.000^***^
	NC	−0.05	0.045^*^
ES (*R*^2^ = 0.045)	PC	0.48	0.000^***^
	NC	0.11	0.000^***^
	PC	−0.20	0.000^***^

*PC, positive coping; NC, negative coping; SWB, subjective well-being; CR, cognitive reappraisal; ES, expression suppression*.

* *p < 0.05*,

*** *p < 0.001*.

### Structural Equation Modeling

According to the results of the regression analysis, the hypothesis of this study had been preliminarily verified. However, this study also hopes to explore the interaction relationship of specific dimensions and the pair-to-pair correlation among multiple variables. Therefore, it is more effective to use the structural equation model to verify the reasonably *a priori* relationship between variables (Wu, 2013). Before establishing the model, the theoretical test and CFA test should be carried out on a one-dimensional scale. If the original fitted model is not excellent, reconstruction should be carried out. After inspection, χ^2^ = 5736.261, *df* = 1,409, χ^2^/*df* = 4.07, NFI = 0.789, NNFI = 0.823, CFI = 0.832, TLI = 0.823, RMSEA = 0.052. In this study, the items were deleted according to the mi correction index within the modification. The optimized structure fitted well [χ^2^ = 2858.044, *df* = 839, χ^2^/*df* = 3.406, normed fit index (NFI) = 0.868, NNFI = 0.895, CFI = 0.903, TLI = 0.895, RMSEA = 0.046, confidence interval (CI) = 0.044–0.048]; all factor loads were significant (all coefficients were >0.4, except two items' coefficients on positive coping, which were 0.491 and 0.496; the rest were > 0.5, *p* < 0.001). These indexes indicate that the observed variables reflect the potential variables observed and can be used for subsequent structural equation analysis.

**Figure 5:**
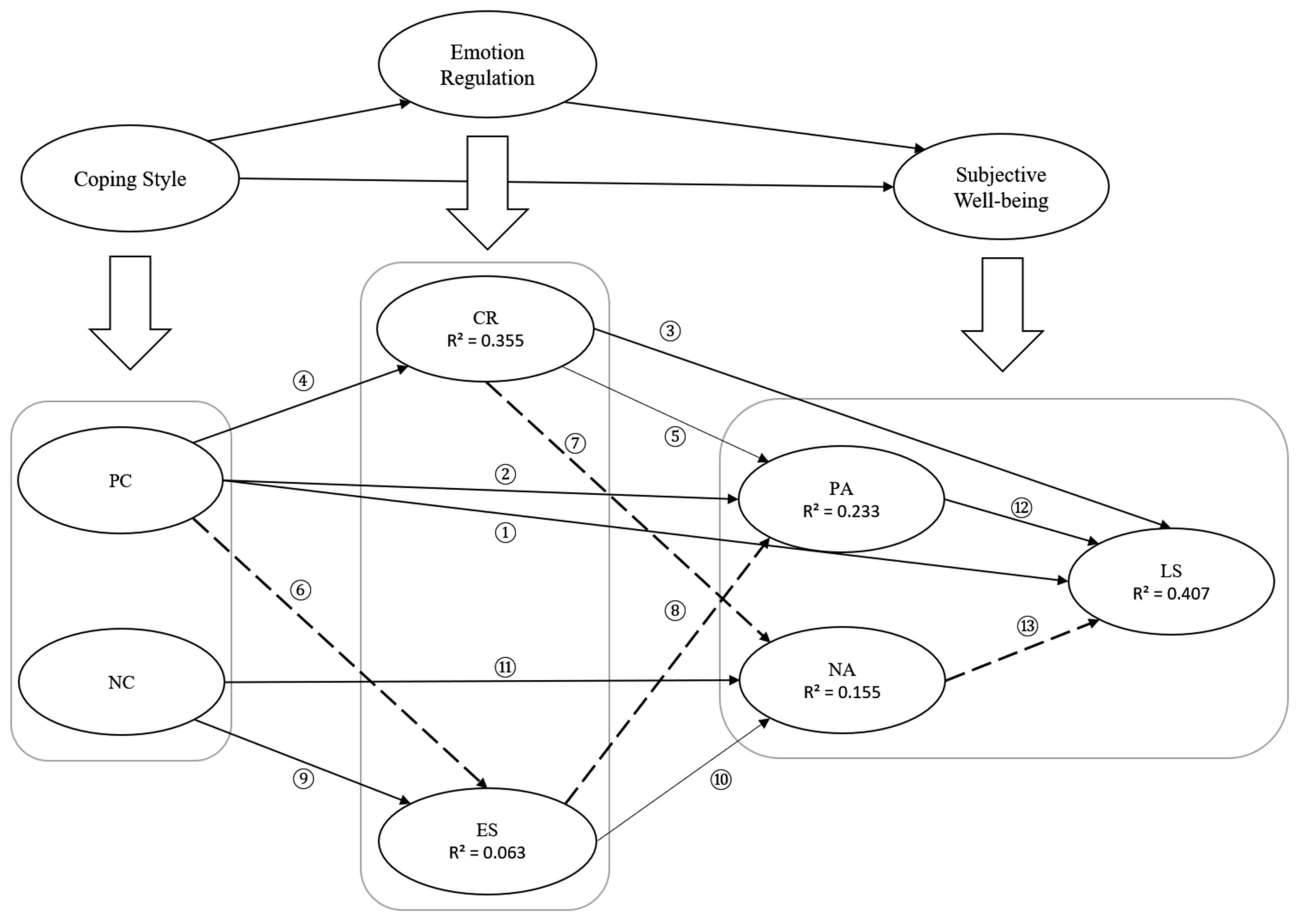
The final structural model for the relationships among coping styles, emotion regulation, and SWB; all the lines shown in the figure were significant (*p* < 0.05); the full lines meant the positive effect, and the imaginary lines meant the negative effect; the path number corresponded to Table 4. PC, positive coping; NC, negative coping; CR, cognitive reappraisal; ES, expression suppression; PA, positive affect; NA, negative affect; LS, life satisfaction; SHAP, SHapley Additive exPlanation.

Before model fitting, the direct predictive effects of independent variables and dependent variables of the mediating model were tested, and the regression analysis of coping style on SWB was conducted. The results showed that the predictive effects were significant (positive coping: *β* = 0.518, *p* < 0.001, negative coping: *β* = −0.138, *p* < 0.001, regression equation *R*^2^ = 0.266, *p* < 0.001). According to the previous assumptions, structural equation model fitting was conducted on the data. Through mi correction index, it is found that there is a covariant relationship between some observation indexes, so the model is modified according to the correction index and theory. According to the standard proposed by Wen Zhonglin (Zhonglin et al., 2004), revised model in this study, χ^2^ = 1668.530, *df* = 816, CFI = 0.959, TLI = 0.955, NFI = 0.923, NNFI = 0.955, incremental fitting index (IFI) = 0.959, standardized root mean square residual (SRMR) = 0.048, RMSEA = 0.030, CI = 0.028–0.033, adjusted goodness of fit index (AGFI) = 0.985, GFI = 0.988, relative fitting index (RFI) = 0.915 (Hu and Bentler, 1998). All the fitting coefficients are excellent, which indicates that the model fits well, and some of the internal path coefficients are not significant (negative coping → cognitive reappraisal, *β* = −0.006, *p* = 0.073, negative coping → life satisfaction, *β* = 0.04, *p* = 0.203, expression suppression → life satisfaction, *β* = −0.03, *p* = 0.246). In order to make the model more concise, we try to delete the insignificant path and found that χ^2^ = 1674.128, *df* = 819, CFI = 0.959, TLI = 0.954, NFI = 0.923, NNFI = 0.954, IFI = 0.959, SRMR = 0.048, RMSEA = 0.030, CI = 0.028–0.033, AGFI = 0.985, GFI = 0.988, RFI = 0.915. In those two models, Δχ^2^/ Δ *df* = 1.866, *p* > 0.05. The simplified model was identified as the optimal model was no different from the original model after deletion. The specific standardized coefficients and *R*^2^ are shown in Figure 5 and Table 4.

**Table 4:** Parameter description of paths in structural equation modeling (SEM).

**Path number**	**Path**		**β**	**95% CI**	
	**Start**	**End**		**Lower**	**Upper**
1	PC	LS	0.11	0.02	0.19
2	PC	PA	0.39	0.31	0.46
3	CR	LS	0.08	0.01	0.15
4	PC	CR	0.58	0.53	0.63
5	CR	PA	0.08	0.00	0.16
6	PC	ES	−0.20	−0.27	−0.12
7	CR	NA	−0.19	−0.25	−0.13
8	ES	PA	−0.14	−0.21	−0.07
9	NC	ES	0.15	0.07	0.24
10	ES	NA	0.09	0.02	0.16
11	NC	NA	0.31	0.24	0.39
12	PA	LS	0.42	0.36	0.48
13	NA	LS	−0.31	−0.36	−0.25

*The path number corresponded to Figure 4. PC, positive coping; NC, negative coping; PA, positive affect; NA, negative affect; LS, life satisfaction; CR, cognitive reappraisal; ES, expression suppression*.

### Test Model Rationality

A bootstrap of deviation correction was adopted to test the model's rationality and mediating effect (Yuan et al., 2007). Three thousand bootstrap samples were taken from the sample (*N* = 1,127) of this study for indirect effect estimation, while the mediating effect is significant as all the 95% CIs do not contain 0 (Fang and Zhang, 2012). This study found that emotion regulation did play a mediating role in the way of coping and SWB. Positive coping affected college students' life satisfaction through two indirect paths: the mediating role of emotion regulation and the chain mediating roles of both emotion regulation and positive affect/negative affect. The negative way of coping does not affect negative affect through cognitive reappraisal, nor does it affect life satisfaction directly by expression suppression and negative affect. Life satisfaction can only affect life satisfaction through the two complete mediators of positive affect or negative affect, whereas cognitive reappraisal can affect life satisfaction through the mediators of positive affect and negative affect. And the total effect of negative coping to life satisfaction is smaller than that of positive coping to life satisfaction.

## Discussion and Conclusion

The current study's main objective was to analyze the relationships among coping styles expressed in either cognitive or behavioral form; emotion regulation strategies used to reduce, maintain, or increase either positive or negative emotions; and SWB. Understanding these relationships may help improve the adjustment process among college students. Specifically, we wanted to find whether emotion regulation strategies mediated the relationship between coping styles and the positive and negative affect of SWB. As hypothesized, according to the significant relationships in the structural model, we found that the more positive an individual's coping style, the more use of cognitive reappraisal, the more positive affect, and less negative affect were reported. In contrast, the more negative an individual's coping style is, the more likely they are to use expression suppression, the more negative effect they report, and the lower life satisfaction they respond to. These results extend previous studies and provide more comprehensive evidence that emotion regulation strategies represent a mechanism that explains the positive relationship between coping styles and SWB.

We found that positive coping was positively associated with SWB. Besides, positive coping appears to influence SWB by first increasing the use of cognitive reappraisal positively. The findings of the positive relationship between positive coping, cognitive reappraisal, and positive affect and life satisfaction are consistent with previous studies' results (Gross and John, 2003; Karademas, 2007; Haga et al., 2009; McRae et al., 2012). People who had positive coping styles were more likely to use a positive way to deal with problems by reframing a stressful situation. They were more likely to adapt to stress by cognitive reappraisal, avoid negative emotions, shape which emotions were experienced, and either augment or diminish an emotion that is currently being experienced (Campos et al., 1994; Gross and Thompson, 2007). Positive coping styles enabled people to appraise a stressful situation more positively by using deeply held values to interpret stress positively. Positive coping may encourage engagement in a proactive process that promotes health and well-being. Our findings provide additional support for the growing literature indicating that coping styles play an essential role in the psychosocial adjustment of youth development and, subsequently, their quality of life.

Negative coping was found to have a positive association with both expression suppression and negative affect. However, negative coping failed to predict SWB directly in this study, which is inconsistent with prior studies (Turner et al., 2000; Wollaars et al., 2007; Anshel and Brinthaupt, 2014). There were two possible explanations anticipated for the non-significant relationships. One possible explanation of this result was that in our study, the negative coping measure focused exclusively on avoiding thoughts of the stressor by seeking distraction, whereas the positive coping measure focused on sharing feelings with relatives and friends, being proactive, and solving the problem. However, the results showed that Chinese culture emphasizes family and relationship-based support (Shen and Yeatts, 2013), and Mak and Ho (2007) suggested that relationship-focused positive coping styles are essential during stressful periods.

Therefore, negative coping styles may not significantly impact Chinese college students' SWB than family and relationship-based positive coping styles. Another possible interpretation for the limited connections between negative coping and SWB in the present study is that the populations examined in previous studies include patients and police officers, whereas the current study focused on college students. The levels of stress among patients (Turner et al., 2000; Wollaars et al., 2007) and police officers (Anshel and Brinthaupt, 2014) are high. Stress was influenced by anxiety and depression (Garlow et al., 2008). Stress may be a moderator of the relationship between coping style and SWB. Negative coping strategies may reduce psychological distress in patients and relieve the amount of job stress among police officers. However, most college students have little pressure outside of their studies. When they sometimes encounter a stressful event, they tend to use positive coping styles to share with friends or seek ways to solve it.

The findings that cognitive reappraisal was positively associated with SWB get support in Diener and colleagues” extensive work on SWB. Cognitive reappraisal, in particular, has been consistently found to be strongly correlated with measures of SWB and life satisfaction (e.g., Diener and Emmons, 1984; Diener and Diener, 1995). A recent finding was that countries with more individualistic cultures show stronger correlations between cognitive reappraisal and life satisfaction (Diener and Diener, 1995). The importance of cognitive reappraisal therefore appears to vary across cultures. In addition, previous results showed that suppression has been linked with lower life satisfaction (Gross and John, 2003) and poorer social outcomes, such as increased negative affect and decreased positive affect (Butler et al., 2003; Srivastava et al., 2009). The results of the present study were in line with these results.

In summary, a good fit between the proposed model and the data was indicated by the structural equation modeling analysis. Besides, the predictor variables' structural relationship and the dependent variable in the causal model appeared to be consistent with previous research findings. Positive coping appeared to influence SWB by first influencing cognitive reappraisal. However, negative coping appeared to influence SWB by first influencing expression suppression. This relationship has important implications for counseling individuals.

### Implications for Theoretical and Practice

Despite the shortcomings, the present study still has a specific significance. First, college students' SWB should be studied using the dimensions of life satisfaction or positive and negative emotion and a larger framework, including coping styles and emotion regulation. Second, never should the importance of the mediating effect of emotion regulation in improving college students' psychological health be ignored. Psychologists may engage in studies to suggest college students more to maintain positive coping styles. The need for more extensive positive interventions and prevention is also emphasized (Ash and Huebner, 2001).

Previous studies can summarize that when individuals seek social support or engage in problem solving, this positively affects the SWB of college students markedly (Shek, 1998). Therefore, learning environments to regulate college students' coping styles should be organized by school psychologists, teachers, and school administrators, while offering social support to college students to support their coping resource is available as parents, school psychologists, and school administrators, thus increasing students' SWB. For example, school psychological counseling and guidance programs may include developing positive emotion regulation strategies and regulating coping styles. According to the present study, Chinese colleges and universities should strengthen mental health education and establish a system of mental health education program system, help university students learn how to deal with stress in a positive way, and introduce more effective emotion regulation strategies to students.

The present study has provided new knowledge of how different coping styles affect the emotion regulation strategies and SWB of college students. And theoretically, positive coping and negative coping styles distinctly have differing importance for emotion regulation and well-being. The findings have important implications for coping styles (Ockhuijsen et al., 2014) and well-being promotion programs (Gazmararian et al., 2014). Such programs enable to improve SWB, as well as enhance effectively coping styles, thus reducing stress among college students. Focusing on positive outcomes of different coping strategies recently enjoyed enormous popularity while recognizing that positive coping should enhance positive aspects of well-being. Coping styles showing this effect have generally been studied individually or have not been explicitly differentiated. Because the different coping strategies rely on different mechanisms, research examining their differences is critical for theoretical development as research examining their similarities. After offering the first step, more studies have followed hopefully.

### Limitation

Even though this study provides findings consistent with others, it has several limitations. First, the present study is limited by the static, single point in college students' assessment of coping style, emotion regulation, and SWB. Applied to the cross-sectional approach, it is impossible to establish the causal role of coping style or emotion regulation. To clarify the dynamics of the links between coping style, emotional regulation, and SWB, it would be more suitable to assess them as they change over time in a longitudinal and sequential design. The sample in our study nearly all came from university students. One reason is that our research topic is focused on positive youth development, as there are growing mental health problems among Chinese youths (Lo et al., 2018; Shek and Siu, 2019), thus seeking effective emotional regulation strategies and coping styles to improve SWB particularly important. In future study, it will be critical to examine whether the current findings generalize to other age groups and other cultural contexts.

Second, our study focused on SWB. The other key construct of well-being, PWB, has been neglected. Although both constructs are highly correlated, they address a distinct facet of overall well-being: SWB has been operationally defined as high life satisfaction combined with high levels of positive affect and low levels of negative affect (Diener and Emmons, 1984; Lucas et al., 1996), whereas PWB has been operationalized by a six-dimensional framework comprising positive relations, autonomy, environmental mastery, personal growth, purpose in life, and self-acceptance (Ryff and Keyes, 1995; McGregor and Little, 1998; Ryan and Deci, 2001). Keyes' research brings SWB and PWB together, showing that they do not simply involve overlapping distributions but rather lead to characterizations of genuinely distinct forms of what it means to be well, and they proposed that SWB and PWB may also compensate for each other (Keyes et al., 2002). Future studies will provide more conclusive evidence, in which we seek to relate coping styles or emotion regulation strategies not to SWB and PWB as separate domains but to their combinations.

In addition, self-report surveys are relied on by most research of SWB containing some biases, despite that there is some convergence shown by some reports. Accordingly, it is essential to examine different methods' unique predictive values. Whether the findings enable replication by other types of measures of SWB is still a question. Fourth, genes play an essential role in SWB, and more studies should consider biological mechanisms genetically. Finally, outcomes (e.g., earning and stable romantic relationships) across individuals and cultures were varied by optimal levels of SWB. So, identifying the systematic individual and cultural variations in optimal levels of SWB is critical.

### Further Research

This study provides a model for understanding the relationship among positive coping and negative coping, emotion regulation, and SWB among college students. Developing this field needs to refine and validate this model to provide important information by replicating research with “big data” among various individuals' diversity. This model should be verified by the studies with multiple instruments and observation, thus providing a more accurate assessment of these constructs. In conclusion, verifying further clarification of the interrelationship among coping, emotion regulation, and SWB is available by more replicating research, thus facilitating the ongoing development of practical assessment and treatments to improve youth development's psychosocial functioning.

## Data Availability Statement

The raw data supporting the conclusions of this article will be made available by the authors, without undue reservation.

## Ethics Statement

The studies involving human participants were reviewed and approved by The local Ethics Committee of Henan University. The patients/participants provided their written informed consent to participate in this study.

## Author Contributions

YC and XJ designed the study and drafted the manuscript. XJ and LJ performed the study. CZ, CG, XZ, and LJ analyzed the data and editing of the paper. YC revised the paper. All authors read and approved the final manuscript.

## Conflict of Interest

The authors declare that the research was conducted in the absence of any commercial or financial relationships that could be construed as a potential conflict of interest.
